# Endometrial Polyps and Subfertility in Women Under 40: Pathophysiology, Fertility Outcomes, and Clinical Management

**DOI:** 10.3390/medicina62040692

**Published:** 2026-04-03

**Authors:** Goksu Goc, Ozer Birge

**Affiliations:** 1 Department of Obstetrics and Gynecology, American Hospital Kosovo, 10000 Prishtine, Kosovo; 2 Department of Gynecology and Obstetrics, Eskişehir City Hospital, Eskişehir 26080, Türkiye; ozbirge@gmail.com

**Keywords:** endometrial polyps, infertility, subfertility, hysteroscopy, hysteroscopic polypectomy, intrauterine insemination, in vitro fertilization, endometrial receptivity, implantation

## Abstract

*Background and Objectives*: Endometrial polyps are common in women presenting with subfertility, yet uncertainty persists regarding which lesions warrant removal and how best to integrate hysteroscopic management with contemporary fertility treatment pathways. This narrative review synthesizes current evidence on pathophysiological mechanisms, diagnostic approaches, fertility outcomes, and practical clinical management for women under 40 years of age. *Materials and Methods*: PubMed/MEDLINE, Embase, Scopus, Web of Science, and the Cochrane Library were searched for English-language human studies published between January 2005 and December 2025. From 2352 records identified, 83 studies were included after screening of 1517 unique records (7 randomized controlled trials, 12 systematic reviews/meta-analyses, 14 prospective cohort studies, 31 retrospective cohort studies, 5 case–control and other study designs, 11 narrative reviews and supporting evidence studies, 1 clinical guideline, and 2 targeted 2025 additions). This structured narrative review employed a systematic search strategy to ensure comprehensive coverage, with evidence synthesized thematically in accordance with the SANRA guidelines. No formal risk-of-bias assessment or pre-registered protocol was used. *Results*: Across treatment modalities, hysteroscopic polypectomy was consistently associated with improved fertility outcomes. The landmark Pérez-Medina randomized trial reported a relative risk of 2.1 (95% CI 1.5–2.9) for pregnancy after polypectomy before intrauterine insemination. For IVF/ICSI, reported clinical pregnancy rates after polypectomy range from 53–72% and live birth rates from 43–66%. Proposed mechanisms include mechanical interference, chronic inflammation with cytokine dysregulation, altered endometrial receptivity (including dysregulation of HOXA10/HOXA11), and impaired decidualization. *Conclusions:* Current evidence supports hysteroscopic polypectomy as an effective intervention to improve fertility outcomes in subfertile women with endometrial polyps, particularly prior to intrauterine insemination. For IVF/ICSI, polypectomy of documented polyps appears beneficial, though evidence quality is moderate and heterogeneity exists across studies. It is critical to distinguish routine screening hysteroscopy before IVF from targeted polypectomy when a polyp has been documented. Contemporary guidance (including the 2024 SOGC guideline) favors polypectomy for symptomatic polyps and those that meet specific clinical criteria; for small asymptomatic polyps (<10 mm), individualized decision-making is appropriate, given limited direct evidence and the potential for spontaneous regression. Future research should clarify molecular predictors of polyp-associated infertility, optimal timing relative to fertility treatment, and long-term reproductive outcomes.

## 1. Introduction

Endometrial polyps are localized overgrowths of endometrial tissue that project into the uterine cavity, consisting of endometrial glands, stroma, and blood vessels covered by epithelium [1]. These benign lesions are among the most common intrauterine abnormalities encountered in clinical practice, with prevalence estimates ranging from 7.8% to 34.9%, depending on the population studied and diagnostic methods employed [2,3]. In the general population, endometrial polyps are identified in approximately 10–15% of women, while their prevalence increases substantially to 16–32% among women presenting with subfertility [4,5].

Historical understanding of endometrial polyps and their relationship to fertility has evolved considerably. Early case reports from the 1950s and 1960s suggested a possible association between intrauterine lesions and reproductive failure, but systematic evaluation became feasible only with modern hysteroscopy in the 1970s. The introduction of office hysteroscopy in the 1990s revolutionized polyp diagnosis, allowing direct visualization without general anesthesia [6]. The landmark randomized controlled trial by Pérez-Medina and colleagues in 2005 represented a watershed moment in the field, providing level I evidence that hysteroscopic polypectomy before intrauterine insemination significantly improves pregnancy rates [7]. This study fundamentally changed clinical practice and established polypectomy as a standard intervention in fertility centers worldwide.

The clinical significance of endometrial polyps in reproductive medicine extends beyond their direct impact on conception rates. Infertility affects a substantial proportion of couples globally, representing a significant public health burden with considerable economic and psychological consequences [7]. When endometrial polyps are identified in subfertile women, they present a potentially treatable cause of reproductive failure. The economic implications are considerable: assisted reproductive technology (ART) cycles cost $12,000–$15,000 per attempt in the United States, and improving baseline fertility potential through polyp removal may reduce the number of cycles needed to achieve pregnancy [8].

The relationship between endometrial polyps and subfertility has been a subject of considerable clinical interest. While the association between these lesions and impaired fertility is well-documented, establishing a direct causal relationship remains challenging due to the multifactorial nature of infertility and frequent coexistence of other reproductive pathologies [9]. Nevertheless, accumulating evidence suggests that endometrial polyps may interfere with fertility through multiple mechanisms, including mechanical obstruction of sperm transport, disruption of embryo implantation, and alteration of the endometrial microenvironment [7,10].

Despite substantial research progress, several important controversies persist. The management of small asymptomatic polyps (<10 mm) remains contentious. While some clinicians advocate for routine removal of all polyps in subfertile women, others suggest expectant management for small lesions, citing the potential for spontaneous regression [11,12]. The 2024 Society of Obstetricians and Gynaecologists of Canada (SOGC) guideline issued a strong recommendation for polypectomy in subfertile women, including small polyps [13]. However, this recommendation is based largely on extrapolation from the Pérez-Medina trial [7] and observational data rather than on randomized evidence specifically addressing polyps <10 mm. Given that spontaneous regression occurs in 25–27% of small polyps [11,12] and no dedicated trial has demonstrated fertility benefit from removing asymptomatic polyps <10 mm, the strength of this recommendation for the smallest lesions remains debated.. The optimal timing of surgical intervention relative to fertility treatment also lacks consensus. Polyp recurrence is another clinical challenge, with reported rates ranging from 3.7% to 15% [14].

This narrative review synthesizes current evidence on endometrial polyps and subfertility in women under 40 years of age. We examine pathophysiological mechanisms, diagnostic approaches, treatment outcomes across different fertility modalities, and evidence-based recommendations for clinical management. Key studies are summarized in Table 1.

## 2. Materials and Methods

This study was designed as a structured narrative review with a systematic search strategy. A systematic search was employed to ensure comprehensive identification of relevant literature, while a narrative synthesis approach was adopted given the heterogeneity of study designs, populations, and outcomes, which precluded formal meta-analytic pooling. The review was conducted in accordance with the SANRA guidelines [20].

### 2.1. Search Strategy

Five electronic databases were systematically searched: PubMed/MEDLINE (National Library of Medicine, Bethesda, MD, USA; https://pubmed.ncbi.nlm.nih.gov, accessed on 15 March 2025) (*n* = 612), Embase (Elsevier, Amsterdam, The Netherlands; https://www.embase.com, accessed on 15 March 2025) (*n* = 534), Scopus (Elsevier, Amsterdam, The Netherlands; https://www.scopus.com, accessed on 15 March 2025) (*n* = 489), Web of Science Core Collection (Clarivate Analytics, Philadelphia, PA, USA; https://www.webofscience.com, accessed on 15 March 2025) (*n* = 427), and Cochrane Library (John Wiley & Sons, Hoboken, NJ, USA; https://www.cochranelibrary.com, accessed on 15 March 2025) (*n* = 212). Additional records were identified through reference list screening (*n* = 52) and citation searching (*n* = 26), yielding a total of 2352 records. The search covered publications from January 2005 to December 2025. Search terms included combinations of: endometrial polyp, uterine polyp, intrauterine polyp, subfertility, infertility, fertility, pregnancy, conception, IVF, ICSI, IUI, assisted reproduction, and hysteroscop.

### 2.2. Inclusion and Exclusion Criteria

The initial search yielded 2352 records. After removing duplicates, 1517 unique records were screened based on title and abstract. Full-text articles were retrieved for 267 potentially eligible studies. Following a detailed review, 186 articles were excluded for the following reasons: not addressing fertility outcomes (*n* = 67), postmenopausal populations (*n* = 34), case reports with fewer than 10 patients (*n* = 28), non-English language (*n* = 23), conference abstracts (*n* = 18), and other reasons (*n* = 16). 81 studies met the inclusion criteria from the database search. Together with the two targeted 2025 additions (Pîrlog et al. 2025 [21] and Wang et al. 2025 [22]), 83 studies were included in the final synthesis. The study selection process is illustrated in the PRISMA flow diagram (Supplementary Material S3).

### 2.3. Study Selection and Data Extraction

The final analysis included 83 studies: 7 randomized controlled trials, 12 systematic reviews and meta-analyses, 14 prospective cohort studies, 31 retrospective cohort studies, 5 case–control and other study designs, 11 narrative reviews and supporting evidence studies, and 1 clinical guideline and 2 targeted 2025 additions. Data were independently extracted by both authors using a standardized form, with discrepancies resolved by consensus. The complete included-study list with summary data is provided in Supplementary Table S1. Data were synthesized thematically using a narrative approach.

### 2.4. Quality Assessment

This narrative review followed the Scale for the Assessment of Narrative Review Articles (SANRA) guidelines [20]. The systematic search methodology and transparent reporting of screening results (including a PRISMA-style flow diagram) were adopted to enhance reproducibility and minimize selection bias, while the narrative synthesis framework was chosen to accommodate the clinical breadth and methodological diversity of the included literature. Given the narrative synthesis approach and the inclusion of diverse study designs, formal risk-of-bias assessment using tools such as the Cochrane Risk of Bias tool or Newcastle–Ottawa Scale was not performed. However, study quality considerations (including sample size, prospective vs. retrospective design, presence of control groups, and adjustment for confounders) were incorporated into the interpretation of findings. The SANRA assessment checklist is provided in Supplementary Material S4.

## 3. Epidemiology and Risk Factors

The epidemiology of endometrial polyps varies significantly depending on the population studied and diagnostic methods employed. In the general female population, prevalence estimates range from 7.8% to 34.9%, with most studies reporting rates of 10–15% [2,23]. Among women presenting with subfertility, prevalence increases substantially to 16–32%, suggesting either a causal relationship or shared risk factors [4,5]. The prevalence increases with age, reaching approximately 8% by age 40 and continuing to rise through the perimenopausal period [23].

Several risk factors have been identified for the development of endometrial polyps. Obesity represents a significant risk factor, with body mass index (BMI) > 30 kg/m^2^ associated with increased polyp prevalence, likely mediated through elevated peripheral estrogen conversion in adipose tissue [23]. Hypertension has been consistently associated with polyp formation, though the mechanism remains unclear. Tamoxifen use in breast cancer patients dramatically increases polyp risk, with prevalence rates of 30–60% reported in this population [13]. Other identified risk factors include nulliparity, late menopause, and hormone replacement therapy use.

Geographic and ethnic variations in polyp prevalence have been observed, though these may partly reflect differences in screening practices and diagnostic thresholds. Studies from Europe report prevalence rates of 15–25% in subfertile populations, while Asian studies report slightly lower rates of 12–20% [23].

## 4. Pathophysiology of Polyp-Associated Subfertility

**Figure 1:**
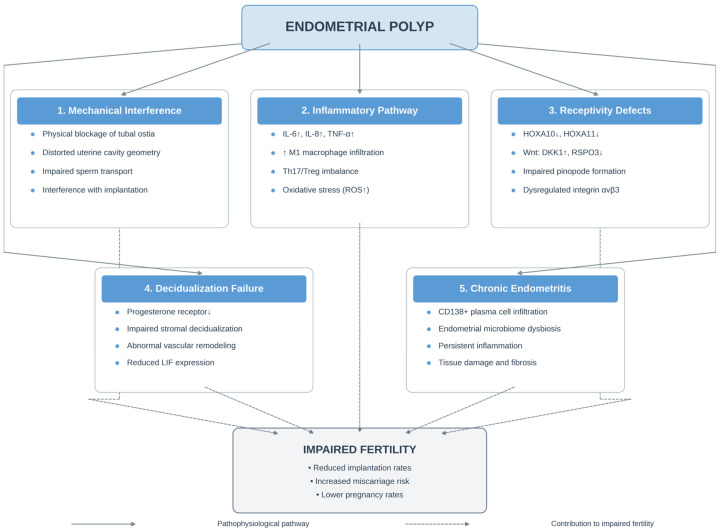
Integrated pathophysiological mechanisms linking endometrial polyps to subfertility. The diagram illustrates multiple interconnected pathways by which endometrial polyps impair fertility, including: (1) mechanical interference with sperm transport and embryo implantation; (2) intrinsic molecular abnormalities including dysregulated Wnt signaling and vascular defects; (3) chronic inflammation with altered cytokine profiles; (4) impaired endometrial receptivity and HOXA gene dysregulation; and (5) disruption of the immune microenvironment and decidualization. These mechanisms act synergistically to reduce fertility potential. ↑ indicates upregulation or increased expression; ↓ indicates downregulation or decreased expression.

The mechanisms by which endometrial polyps impair fertility are multifactorial and involve mechanical, inflammatory, and molecular pathways. Understanding these mechanisms is essential for rational clinical decision-making (Figure 1).

### 4.1. Mechanical Interference and Anatomical Disruption

Endometrial polyps can physically obstruct the reproductive tract at multiple levels. Large polyps may occlude the tubal ostia, preventing sperm from reaching the fallopian tubes for fertilization. Even small polyps can impede sperm transport through the uterine cavity by creating physical barriers or altering normal flow patterns of uterine fluid [7,9]. Additionally, polyps may mechanically prevent embryo implantation by occupying potential implantation sites or creating an irregular endometrial surface that impairs embryo-endometrial apposition. The location of polyps appears to influence their impact on fertility, with fundal polyps potentially having greater effects on implantation than those in the lower uterine segment [24].

### 4.2. Molecular and Transcriptomic Abnormalities

Recent transcriptomic analyses have revealed intrinsic molecular abnormalities within endometrial polyps that extend beyond simple mechanical effects. Chiu et al. (2024) performed RNA sequencing on 12 paired samples of endometrial polyps and adjacent normal endometrium from infertile women, identifying 322 differentially expressed genes [25]. Protein–protein interaction network analysis revealed significant dysregulation in two key pathways: Wnt signaling and vascular smooth muscle regulation.

Specifically, Wnt-related genes showed complex alterations: DKK1 and DKKL1 (Wnt antagonists) were upregulated, while GPC3, GREM1, RSPO3, SFRP5, and WNT10B were downregulated [11]. This pattern suggests dysregulated Wnt signaling that may promote unrestrained growth and impair normal endometrial differentiation. Nearly all genes related to vascular smooth muscle contraction were downregulated in polyps, including ACTA2, ACTG2, KCNMB1, KCNMB2, MYL9, PPP1R12B, and TAGLN [11]. These vascular defects may contribute to abnormal bleeding patterns and impaired endometrial perfusion.

### 4.3. Chronic Inflammation and Cytokine Dysregulation

Endometrial polyps are frequently associated with chronic inflammation, characterized by increased infiltration of immune cells (macrophages, lymphocytes, and plasma cells) and altered cytokine profiles [15,26]. Studies have demonstrated elevated levels of pro-inflammatory cytokines in polyp tissue and surrounding endometrium, including interleukin-6 (IL-6), interleukin-8 (IL-8), and tumor necrosis factor-alpha (TNF-α) [16,18].

This inflammatory milieu may impair fertility through multiple mechanisms. Pro-inflammatory cytokines can disrupt normal endometrial receptivity by interfering with the expression of adhesion molecules and growth factors required for embryo implantation [27]. IL-6 and TNF-α have been shown to inhibit trophoblast invasion and reduce expression of leukemia inhibitory factor (LIF), a critical cytokine for implantation [28,29].

Chronic endometritis (CE), defined by the presence of plasma cells in the endometrial stroma, is detected in 14–57% of women with endometrial polyps [12,30]. The coexistence of polyps and CE may represent a synergistic impairment of fertility, with both conditions contributing to chronic inflammation and altered endometrial function. The diagnosis and management of chronic endometritis in women with polyps is discussed in detail in Section 7.

### 4.4. Altered Endometrial Receptivity and HOXA Gene Dysregulation

Endometrial receptivity, the transient window during which the endometrium is permissive to embryo implantation, is regulated by a complex network of genes and signaling pathways [31]. Several studies have demonstrated that endometrial polyps are associated with altered expression of receptivity markers, both within the polyp tissue itself and in the surrounding endometrium [32,33].

HOXA10 and HOXA11, members of the homeobox gene family, play critical roles in endometrial development, decidualization, and implantation [34]. Pîrlog et al. (2025) reviewed evidence linking altered HOXA10/HOXA11 expression to benign endometrial disorders, including polyps [21,34,35]. HOXA gene dysregulation may perturb endometrial receptivity through multiple mechanisms: impaired endometrial differentiation during the secretory phase, reduced expression of implantation-related genes (including integrin αvβ3 and HOXA10-regulated targets), and altered decidualization in response to progesterone [21,36].

Studies using endometrial receptivity arrays (ERAs) have shown that women with endometrial polyps have a higher prevalence of displaced implantation windows than controls [37]. This suggests that polyps may cause temporal dysregulation of endometrial receptivity, such that the window of maximal receptivity does not align with the expected timing of embryo arrival or transfer.

Other receptivity markers altered in the presence of polyps include: reduced expression of pino-podes (ultrastructural markers of receptivity) [38], decreased glycodelin and osteopontin (secretory proteins involved in implantation) [6,39], and altered expression of progesterone receptor isoforms [40]. These molecular changes may extend beyond the polyp itself to affect the entire endometrial cavity, providing a mechanistic explanation for why even small polyps can impair fertility.

### 4.5. Impaired Decidualization and Immune Microenvironment

Decidualization, the progesterone-driven transformation of endometrial stromal cells into specialized decidual cells, is essential for successful implantation and maintenance of early pregnancy. Endometrial polyps have been associated with impaired decidualization, characterized by reduced expression of decidual markers (prolactin, IGFBP-1) and altered stromal cell morphology [41,42].

The endometrial immune microenvironment plays a critical role in implantation and early pregnancy. The normal mid-secretory endometrium contains a balanced population of immune cells, including uterine natural killer (uNK) cells, macrophages, and regulatory T cells, which facilitate trophoblast invasion while maintaining tolerance to the semi-allogeneic embryo [43]. Studies have shown that endometrial polyps are associated with altered immune cell populations, including increased macrophage infiltration and altered uNK cell phenotypes [44,45].

Additionally, polyps may disrupt the normal balance between pro-inflammatory and anti-inflammatory signals required for successful implantation. The “inflammatory paradox” of implantation, whereby a controlled inflammatory response is necessary for trophoblast invasion but excessive inflammation is detrimental, may be disrupted by polyps [46]. This dysregulated immune environment may contribute to implantation failure or early pregnancy loss even after successful initial implantation.

## 5. Diagnostic Approaches

Accurate diagnosis of endometrial polyps is essential for appropriate management. Multiple imaging modalities are available, each with distinct advantages and limitations. The diagnostic characteristics of various modalities are summarized in Table 2.

### 5.1. Transvaginal Ultrasonography

Two-dimensional transvaginal ultrasonography (2D-TVS) is typically the first-line imaging modality for evaluating the endometrium. Polyps appear as hyperechoic focal lesions within the endometrial cavity, often with a feeding vessel demonstrable on color Doppler imaging. However, diagnostic accuracy is limited, with reported sensitivity ranging from 55% to 88% and specificity from 65% to 95% [47,48,49,50]. Performance is optimal during the proliferative phase when the thin endometrium provides better contrast with polyps. Three-dimensional ultrasound (3D-TVS) provides improved visualization of the uterine cavity and may increase diagnostic accuracy to 90–95% [51].

### 5.2. Saline Infusion Sonohysterography

Saline infusion sonohysterography (SIS), also known as sonohysterography, involves instilling sterile saline into the uterine cavity during transvaginal ultrasound. This technique significantly improves polyp detection compared with standard TVS, with sensitivities of 87–94% and specificities of 81–94% [48,52,53,54,55,56,57]. SIS enables more precise delineation of polyp size, location, and relationship to the tubal ostia. The procedure is well-tolerated in the office setting and provides valuable information for surgical planning. Meta-analysis has confirmed the superiority of SIS over 2D-TVS for polyp detection, with a pooled sensitivity of 92% versus 55% [48].

### 5.3. Hysteroscopy

Hysteroscopy remains the gold standard for diagnosis of endometrial polyps, with a sensitivity of 92–100% and a specificity of 75–94% [9,16,53]. Direct visualization allows accurate assessment of polyp characteristics and enables simultaneous treatment. Office hysteroscopy, performed without anesthesia using small-diameter hysteroscopes (≤5 mm), has become increasingly popular due to convenience, cost-effectiveness, and high patient acceptability [6,58,59,60]. Studies report successful completion rates of 95–98% for office procedures, with minimal complications. The “see-and-treat” approach, combining diagnosis and treatment in a single office visit, offers significant advantages in terms of efficiency and patient convenience.

### 5.4. Diagnostic Algorithm

For subfertile women, a stepwise diagnostic approach is recommended. Initial evaluation with TVS during the proliferative phase can identify obvious polyps. When TVS findings are equivocal or polyps are suspected based on clinical presentation, SIS improves accuracy. Hysteroscopy should be performed when imaging suggests polyps requiring treatment or when the diagnosis remains uncertain.

## 6. Surgical Treatment

The impact of hysteroscopic polypectomy on fertility outcomes has been evaluated in multiple studies across different fertility treatment contexts: intrauterine insemination (IUI), in vitro fertilization/intracytoplasmic sperm injection (IVF/ICSI), and natural conception. The quality and consistency of evidence vary by treatment modality.

### 6.1. Intrauterine Insemination

The strongest evidence for the benefit of hysteroscopic polypectomy comes from studies of women undergoing IUI. The landmark randomized controlled trial by Pérez-Medina et al. (2005) randomized 215 infertile women with endometrial polyps to either hysteroscopic polypectomy followed by IUI (n = 107) or diagnostic hysteroscopy alone followed by IUI (n = 108) [7]. The polypectomy group had significantly higher pregnancy rates: 63% (67/107) versus 28% (30/108), yielding a relative risk of 2.1 (95% CI 1.5–2.9, *p* < 0.001) [7]. This trial provides Level I evidence supporting polypectomy before IUI.

Subsequent observational studies have corroborated these findings. Stamatellos et al. (2008) reported pregnancy rates of 78.3% (65/83) after polypectomy in women with unexplained infertility, with most pregnancies occurring within 12 months [10]. Shokeir et al. (2004) found that polypectomy improved pregnancy rates in women with unexplained infertility and polyps, with a relative risk of 2.3 (95% CI 1.1–4.8) compared to expectant management [5]. A 2019 systematic review by Zhang et al. included five studies (one RCT and four cohort studies) comparing polypectomy versus no treatment in women undergoing IUI or attempting natural conception [27]. The pooled analysis showed significantly higher pregnancy rates after polypectomy (OR 2.9, 95% CI 1.8–4.6, *p* < 0.001) [27]. The benefit was consistent across studies despite heterogeneity in polyp size, patient age, and duration of infertility.

### 6.2. In Vitro Fertilization and Intracytoplasmic Sperm Injection

Evidence for the benefit of polypectomy in women undergoing IVF/ICSI is more heterogeneous, though most studies suggest improved outcomes. It is critical to distinguish two clinical scenarios: (1) polypectomy of documented polyps before IVF, and (2) routine screening hysteroscopy before IVF with polypectomy of incidentally detected polyps.

#### 6.2.1. Polypectomy of Documented Polyps Before IVF

Multiple retrospective cohort studies have reported favorable outcomes after polypectomy in women with documented polyps undergoing IVF/ICSI. Reported clinical pregnancy rates after polypectomy range from 53% to 72%, and live birth rates from 43% to 66% [15,16,17,26]. Triantafyllidou et al. (2024) reported outcomes in 40 women with unexplained infertility who underwent hysteroscopic polypectomy before IVF [15]. The clinical pregnancy rate was 65% (26/40), and the total positive pregnancy rate was 72.5% (29/40, including 3 biochemical pregnancies) [15]. This retrospective cohort study demonstrates favorable outcomes after polypectomy in carefully selected patients with unexplained infertility.

Wen et al. (2024) analyzed 1247 infertility patients undergoing IVF/ICSI, of whom 312 had concomitant endometrial polyps [26]. After polypectomy, clinical pregnancy rates and live birth rates were comparable to those of patients without polyps, suggesting that polypectomy normalizes reproductive outcomes [26]. A 2024 systematic review and meta-analysis by Wang et al. evaluated whether hysteroscopy improves fertility outcomes in women undergoing IVF/ICSI [16]. The analysis included 23 studies with 11,887 patients. Hysteroscopy with treatment of identified pathology (including polypectomy) was associated with improved clinical pregnancy rates (RR 1.34, 95% CI 1.19–1.51) and live birth rates (RR 1.43, 95% CI 1.21–1.69) compared to no hysteroscopy [16]. However, this analysis included women with various intrauterine pathologies, not exclusively polyps.

#### 6.2.2. Routine Screening Hysteroscopy Before IVF

The role of routine screening hysteroscopy before IVF in unselected patients remains controversial. The Cochrane review by Bosteels et al. (2018) evaluated the evidence for hysteroscopy as a diagnostic or therapeutic intervention before IVF/ICSI [61]. The review concluded that hysteroscopy may improve clinical pregnancy rates in women with suspected uterine cavity abnormalities, but the overall certainty of evidence was low to moderate, with substantial heterogeneity across included trials. The TROPHY trial (El-Toukhy et al. 2016), a multicenter RCT of 702 women with recurrent IVF failure, found no significant improvement in live birth rates with routine outpatient hysteroscopy before the next IVF cycle (29% vs. 29%, RR 1.00, 95% CI 0.78–1.27) [62]. Based on these findings, routine screening hysteroscopy before a first IVF cycle is not universally recommended. However, targeted hysteroscopy in women with suspected intrauterine pathology on imaging or those with recurrent implantation failure remains a reasonable clinical approach.

#### 6.2.3. Timing of Embryo Transfer After Polypectomy

The optimal interval between polypectomy and embryo transfer remains debated. Dunn et al. (2018) investigated whether early embryo transfer (<30 days) after hysteroscopic polypectomy affects outcomes compared to delayed transfer (≥30 days) [18]. In 389 frozen embryo transfer cycles, early transfer was associated with lower live birth rates (38% vs. 51%, *p* = 0.02) and higher miscarriage rates (26% vs. 13%, *p* = 0.02) [18]. This suggests that a minimum interval of 30 days should be allowed for endometrial healing and normalization of the inflammatory milieu before embryo transfer. However, other studies have not confirmed this finding. Several retrospective analyses found no difference in outcomes between immediate (next cycle) and delayed embryo transfer after polypectomy [63,64]. The discrepancy may relate to differences in polypectomy technique, polyp size, or patient characteristics. Current practice typically allows 1–2 menstrual cycles between polypectomy and embryo transfer, balancing concerns about endometrial healing with the desire to minimize treatment delay.

#### 6.2.4. Polypectomy Technique

Recent studies have compared different hysteroscopic polypectomy techniques. Wang et al. (2025) compared manual hysteroscopic tissue removal devices versus conventional resection in 240 women undergoing IVF [22]. No significant differences were observed in clinical pregnancy rates, live birth rates, or complications between techniques [22]. This suggests that the benefit of polypectomy is independent of the specific technique used, provided complete polyp removal is achieved. Wang et al. (2024) investigated whether pretreatment with long-acting GnRH agonists before hysteroscopic multiple polypectomy improves outcomes [19]. In women with multiple polyps, GnRH agonist pretreatment was associated with improved pregnancy outcomes, possibly through endometrial thinning that facilitates complete polyp visualization and removal [19].

### 6.3. Natural Conception

Several studies have evaluated fertility outcomes after polypectomy in women attempting natural conception without assisted reproductive technology. Stamatellos et al. (2008) reported that 78.3% of women with unexplained infertility and polyps achieved pregnancy within 12 months after polypectomy, compared to historical controls [10]. Most pregnancies occurred within the first 6 months, suggesting that polypectomy rapidly restores fertility potential. Pérez-Medina et al. (2005) included a subset of women who attempted natural conception after randomization to polypectomy versus diagnostic hysteroscopy alone [7]. Pregnancy rates were higher in the polypectomy group across all conception methods (natural, IUI, and IVF), indicating a general fertility-enhancing effect of polyp removal. A 2017 cost-analysis and systematic review by Mouhayar et al. evaluated the cost-effectiveness of hysteroscopic polypectomy prior to infertility treatment [28]. The analysis concluded that polypectomy is cost-effective when performed before IUI or natural conception attempts, given the relatively low cost of the procedure and the substantial improvement in pregnancy rates [28]. For IVF, cost-effectiveness depends on polyp size and patient age, with greater benefit for larger polyps and younger women.

## 7. Chronic Endometritis: Diagnosis and Management

Chronic endometritis (CE) is increasingly recognized as an important cause of subfertility and recurrent implantation failure. CE is defined histologically by the presence of plasma cells in the endometrial stroma, typically identified using immunohistochemistry for CD138 (syndecan-1), a plasma cell marker [65]. The prevalence of CE in subfertile women ranges from 14% to 57%, depending on the population studied and diagnostic criteria employed [12,30,66,67,68].

The relationship between endometrial polyps and chronic endometritis is complex. Some studies suggest that polyps may harbor or promote chronic inflammation, while others propose that CE and polyps are independent conditions that frequently coexist [12]. Regardless of the causal relationship, the presence of both conditions may synergistically impair fertility, and both warrant treatment in subfertile women.

### 7.1. Standardized CD138-Based Diagnosis

Histologic diagnosis of CE has traditionally relied on identification of plasma cells on hematoxylin and eosin (H&E) staining, but this approach has poor sensitivity and substantial inter-observer variability [69]. Immunohistochemistry for CD138 substantially improves diagnostic accuracy and reproducibility [66].

However, significant heterogeneity exists in the CD138 threshold used to diagnose CE. Reported cutoffs range from ≥1 plasma cell per high-power field (HPF) to ≥5 plasma cells per 10 HPFs [70,71]. This variability complicates the interpretation of the literature and comparison across studies.

Recent efforts have focused on standardizing CD138-based diagnosis. De Smet et al. (2024) developed a 5-tier CD138 scoring system (classes 0–3) based on a systematic evaluation of multiple endometrial samples [72]. The scoring system demonstrated good-to-excellent inter-observer agreement (Fleiss’ Kappa 0.722 for 5-tier classification; 0.858 for a 2-tier clinically relevant classification) [72]. This standardized approach may improve diagnostic consistency and facilitate comparison across studies.

Herlihy et al. (2022) prospectively evaluated the prognostic significance of different CD138 thresholds in 200 women undergoing IVF [73]. Plasma cells were detected in 49% of women using a threshold of ≥1 plasma cell per 10 HPFs, but low plasma cell counts (1, 5, or 10 per 10 HPFs) did not predict implantation or live birth rates [73]. This suggests that low-level CD138 positivity may be prevalent in asymptomatic women and may lack clinical significance.

Liu et al. (2022) used a diagnostic threshold of ≥5 CD138+ cells per HPF in a large cohort of 4003 embryo transfer cycles [74]. Women with ≥5 CD138+ cells/HPF who achieved cure after antibiotic treatment had significantly improved reproductive outcomes compared to those with persistent CE [74]. This supports ≥5 CD138+ cells/HPF as a clinically meaningful threshold that identifies women who benefit from treatment.

Clinical synthesis

Current evidence suggests that a threshold of ≥5 CD138+ plasma cells per HPF represents a reasonable diagnostic cutoff that balances sensitivity and specificity while identifying women most likely to benefit from antibiotic treatment [74,75]. Standardized histopathologic scoring systems, such as that proposed by de Smet et al., should be adopted to improve diagnostic reproducibility [72]. Endometrial biopsy for CE diagnosis should be performed during the proliferative phase (days 5–12 of the menstrual cycle) to avoid confounding by menstrual-phase plasma cells [76].

### 7.2. Antibiotic Treatment Protocols

Multiple antibiotic regimens have been used to treat CE in subfertile women, with substantial heterogeneity in drug choice, dose, duration, and route of administration. The most commonly used regimens include:

Doxycycline: 100 mg orally twice daily for 14 days is the most widely used first-line regimen [65,77]. Doxycycline provides broad-spectrum coverage including common endometrial pathogens (Streptococcus, Enterococcus, Escherichia coli, and Ureaplasma) and has good tissue penetration.

Combination regimens: Some protocols use combination therapy, such as doxycycline plus metronidazole (500 mg orally twice daily for 14 days) to provide additional anaerobic coverage [78]. Ciprofloxacin (500 mg orally twice daily for 14 days) has also been used, either alone or in combination [79].

Intrauterine antibiotic infusion: Luncan et al. (2022) compared intrauterine antibiotic infusion versus oral antibiotic therapy in 90 women with CE undergoing IVF [80]. Intrauterine infusion (gentamicin 80 mg + dexamethasone 8 mg in 5 mL saline, administered via intrauterine catheter) achieved a higher cure rate (89% negative test-of-cure) compared to oral combination therapy (doxycycline + metronidazole, 46% cure rate) [80]. This suggests that local intrauterine delivery may increase histologic resolution rates, though this approach requires further validation.

Test-of-cure: Most protocols recommend repeat endometrial biopsy 1–2 months after antibiotic treatment to confirm CE resolution [74,81]. Women with persistent CE despite initial treatment may benefit from alternative antibiotic regimens or extended treatment duration.

### 7.3. Impact on Fertility Outcomes

Multiple studies have demonstrated that successful treatment of CE improves fertility outcomes in women undergoing assisted reproduction. Liu et al. (2022) performed a systematic review and meta-analysis of 12 studies evaluating antibiotic treatment for CE in women with reproductive failures [82]. Women with cured CE had significantly higher ongoing pregnancy/live birth rates (OR 2.85, 95% CI 1.60–5.08) and clinical pregnancy rates (OR 2.31, 95% CI 1.55–3.44) compared to women with persistent CE [82].

Vitagliano et al. (2022) conducted a systematic review and meta-analysis specifically focused on CE in women undergoing IVF [83]. Women with untreated CE had lower ongoing pregnancy/live birth rates (OR 1.97, *p* = 0.02) and clinical pregnancy rates (OR 2.28, *p* = 0.002) compared to women without CE [83]. Importantly, CE cure after antibiotic therapy increased ongoing pregnancy/live birth rates (OR 5.33, *p* < 0.0001) and clinical pregnancy rates (OR 3.64, *p* = 0.0001) [83]. After successful treatment, outcomes were comparable to women without CE, suggesting that antibiotic therapy normalizes reproductive potential [83].

Li et al. (2023) analyzed pregnancy outcomes in 327 women with recurrent implantation failure, of whom 117 (35.8%) had CE [84]. After antibiotic and platelet-rich plasma (PRP) treatment, 70.9% of women achieved CE cure [84]. The CE-cured group had significantly higher clinical pregnancy rates (60.0% vs. 36.4%, *p* < 0.01) and live birth rates (48.6% vs. 27.3%, *p* < 0.05) compared to women with persistent CE [84].

However, important caveats exist. Zhang et al. (2023) reported that women with antibiotic-cured CE undergoing single euploid frozen embryo transfer had higher early pregnancy loss rates compared to women without CE (23.5% vs. 11.8%, *p* = 0.03) [85]. This indicates that histologic cure does not fully normalize early pregnancy outcomes and suggests residual functional alterations in the endometrium after CE treatment [85].

Clinical synthesis

Current evidence supports screening for CE in women with unexplained infertility, recurrent implantation failure, or recurrent pregnancy loss. Diagnosis should be based on standardized CD138 immunohistochemistry with a threshold of ≥5 CD138+ cells/HPF. First-line treatment is doxycycline 100 mg orally twice daily for 14 days, with test-of-cure biopsy 1–2 months after treatment [74,82,83]. Women with persistent CE may benefit from alternative regimens or intrauterine antibiotic infusion [80]. Successful CE cure substantially improves reproductive outcomes and should be achieved before proceeding with embryo transfer (Table 3).

## 8. Clinical Management and Decision-Making

**Figure 2:**
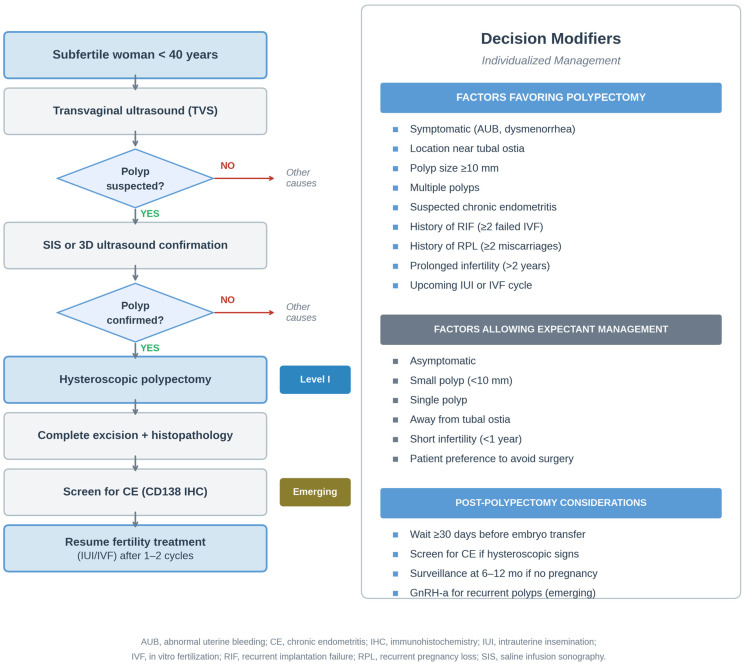
Clinical algorithm for management of endometrial polyps in subfertile women. The flowchart guides clinical decision-making based on polyp characteristics (size, symptoms), patient factors (age, duration of infertility), and planned fertility treatment (expectant management, IUI, IVF/ICSI). Strong evidence supports polypectomy for symptomatic polyps, polyps ≥ 10 mm, and polyps in women undergoing IUI or IVF/ICSI. For small asymptomatic polyps (<10 mm), individualized decision-making is appropriate. Chronic endometritis screening and treatment should be considered in women with hysteroscopic findings suggestive of CE.

Integration of current evidence into clinical practice requires individualized decision-making that considers polyp characteristics, patient age, fertility treatment plan, and patient preferences. The 2024 SOGC guideline recommends hysteroscopic polypectomy for all polyps in subfertile women, regardless of size [13]. While we concur for symptomatic polyps, polyps ≥ 10 mm, and polyps in women undergoing ART, we interpret the current direct evidence as insufficient to support routine removal of all small asymptomatic polyps (<10 mm). The rationale for this more conservative interpretation is outlined below (Figure 2).

### 8.1. Indications for Polypectomy

Strong evidence supports polypectomy in the following scenarios: 1. Symptomatic polyps: Women with abnormal uterine bleeding, dysmenorrhea, or other symptoms attributable to polyps should undergo polypectomy regardless of fertility plans [14]. 2. Polyps ≥ 10 mm: Larger polyps are more likely to impair fertility through mechanical and inflammatory mechanisms and should be removed before fertility treatment [7,27]. 3. Before IUI: Level I evidence supports polypectomy before IUI in women with documented polyps [7]. 4. Before IVF/ICSI with documented polyps: Moderate-quality evidence supports polypectomy of documented polyps before IVF/ICSI [16]. 5. Polyps in women with unexplained infertility: Removal of polyps may restore fertility potential and allow natural conception or less intensive fertility treatment [19,86].

### 8.2. Uncertain Indications

Evidence is less clear for: 1. Small asymptomatic polyps (<10 mm): Limited direct evidence exists for the benefit of removing small polyps, and spontaneous regression occurs in 25–27% of cases [87,88,89]. Individualized decision-making is appropriate, considering patient age, duration of infertility, and planned fertility treatment. 2. Routine screening hysteroscopy before IVF: While some centers perform routine hysteroscopy before IVF to detect and treat unsuspected intrauterine pathology [90], the cost-effectiveness and benefit of this approach remain debated [61,62]. 3. Polyps in women with other identified causes of infertility: When a clear alternative explanation for infertility exists (e.g., severe male factor, tubal occlusion), the incremental benefit of polypectomy is uncertain.

### 8.3. Contraindications and Special Considerations

Hysteroscopic polypectomy is generally safe, with complication rates < 2% [91]. Rare complications include uterine perforation, infection, intrauterine adhesions, and anesthetic complications. Polypectomy should be deferred in women with active pelvic infection or pregnancy.

In women with multiple polyps or polyps > 2 cm, consideration should be given to GnRH agonist pretreatment to thin the endometrium and facilitate complete visualization and removal [19]. Operative hysteroscopy under anesthesia may be required for large or multiple polyps, whereas small, solitary polyps can often be removed during office hysteroscopy without anesthesia.

### 8.4. Post-Polypectomy Management

After polypectomy, most clinicians recommend waiting 1–2 menstrual cycles before proceeding with fertility treatment to allow endometrial healing [18]. However, the optimal interval remains uncertain, and some studies suggest that immediate (next-cycle) treatment is acceptable for small polyps [63,64].

Histopathologic examination of all removed polyps is essential to exclude malignancy or premalignant changes, though the risk is very low in women under 40 years of age (<1%) [92]. If chronic endometritis is suspected based on hysteroscopic appearance (focal hyperemia, stromal edema, micropolyps), endometrial biopsy for CD138 immunohistochemistry should be performed, and antibiotic treatment initiated if CE is confirmed [72,77]. For surveillance of recurrence, routine imaging is not required if complete resection has been confirmed; however, repeat TVS or SIS should be performed at 6–12 months if pregnancy has not been achieved or if symptoms recur. Patients with risk factors for recurrence (multiple polyps, metabolic syndrome, chronic anovulation) should be counseled about modifiable risk reduction and may benefit from closer follow-up.

### 8.5. Expectant Management and Surveillance

For small asymptomatic polyps (<10 mm) in women not immediately pursuing fertility treatment, expectant management with surveillance ultrasound is a reasonable alternative to immediate polypectomy [89]. Spontaneous regression occurs in approximately 25% of small polyps within 12 months [88]. However, if the polyp persists or enlarges, or if the woman subsequently pursues fertility treatment, polypectomy should be performed.

## 9. Discussion

This review synthesizes current evidence on endometrial polyps and subfertility in women under 40 years of age. The findings consistently support hysteroscopic polypectomy as an effective intervention for improving fertility outcomes across treatment modalities.

### 9.1. Strengths of Current Evidence

The evidence base has several notable strengths. First, the Pérez-Medina RCT provides Level I evidence demonstrating a clear benefit of polypectomy before IUI, with a relative risk of 2.1 for achieving pregnancy [7]. The consistency of findings across subsequent observational studies strengthens the generalizability of these results. Second, multiple pathophysiological mechanisms have been identified that link polyps to impaired fertility, thereby providing biological plausibility. Third, recent meta-analyses have confirmed and quantified the benefit of polypectomy [16,27]. Fourth, cost-effectiveness analyses support polypectomy as economically rational [28].

### 9.2. Evidence Gaps and Limitations

Despite the overall strength of evidence, several important gaps warrant acknowledgment. Evidence for polypectomy before IVF is less robust than for IUI, with no large randomized controlled trials directly addressing this question. The management of small polyps (<10 mm) remains uncertain despite recent guideline recommendations. The optimal timing of intervention relative to fertility treatment remains poorly defined.

### 9.3. Clinical Practice Implications

Routine evaluation for endometrial polyps should be incorporated into the diagnostic workup of subfertile women. When polyps are identified, hysteroscopic polypectomy should be considered, although the evidence for removing small asymptomatic polyps (<10 mm) remains limited [13]. Histopathologic examination should be performed to assess for malignancy and chronic endometritis, with antibiotic therapy if plasma cells are identified.

## 10. Special Populations

### 10.1. Advanced Maternal Age

Women of advanced reproductive age (≥35 years) face declining ovarian reserve and potentially increased polyp prevalence. In this population, timely diagnosis and treatment are particularly important. The approach should favor prompt polypectomy with expedited progression to fertility treatment.

### 10.2. Polycystic Ovary Syndrome

Women with polycystic ovary syndrome (PCOS) may have increased polyp prevalence due to chronic anovulation and unopposed estrogen exposure [29]. Management should address both the polyp and underlying PCOS. Metformin and lifestyle modification may reduce the risk of polyp recurrence.

### 10.3. Recurrent Pregnancy Loss

Women with recurrent pregnancy loss should be evaluated for endometrial polyps as part of the comprehensive workup. The association between polyps, chronic endometritis, and RPL suggests that polyp removal and treatment of associated inflammation may reduce miscarriage risk [68].

## 11. Future Directions

Despite substantial progress in understanding the relationship between endometrial polyps and subfertility, several important questions remain unresolved. Not all polyps impair fertility to the same degree. Identification of molecular biomarkers (e.g., specific gene expression signatures, cytokine profiles, or receptivity markers) that predict which polyps are most likely to impair fertility would enable more targeted treatment decisions. The transcriptomic abnormalities identified by Chiu et al. [25] and the HOXA gene dysregulation reviewed by Pîrlog et al. [21] represent promising starting points for such investigations. While most studies suggest waiting 1–2 cycles after polypectomy before embryo transfer, the optimal interval has not been definitively established. Prospective studies comparing immediate versus delayed treatment, with mechanistic endpoints (e.g., endometrial receptivity markers, inflammatory cytokines) in addition to clinical outcomes, would help clarify this question. Most studies have included heterogeneous populations of subfertile women. Subgroup analyses or dedicated studies in specific populations (e.g., women with recurrent implantation failure, recurrent pregnancy loss, or specific polyp characteristics) would refine treatment recommendations. Most studies report short-term outcomes (pregnancy rate, live birth rate in the first treatment cycle). Long-term outcomes, including cumulative live birth rates over multiple cycles, time to pregnancy, and obstetric complications, are less well characterized.

Polyp recurrence rates after hysteroscopic removal range from 3.7% to 15% [93,94]. Identified risk factors include incomplete base resection (particularly with grasping forceps versus resectoscopy), multiple polyps at initial diagnosis, obesity and metabolic syndrome, chronic endometritis, tamoxifen use, chronic anovulation, and older age [93,94]. Technique-specific data suggest that complete base resection using monopolar or bipolar resectoscopy is associated with lower recurrence than blind curettage or simple avulsion [17]. Recommended post-polypectomy surveillance includes: (1) no routine imaging if complete resection was achieved; (2) repeat TVS or SIS at 6–12 months if pregnancy is not achieved; (3) immediate re-evaluation if symptoms recur (abnormal bleeding); and (4) addressing modifiable risk factors (weight management, CE treatment) in high-risk patients. Strategies to prevent recurrence, including hormonal interventions, warrant further investigation.

## 12. Conclusions

Endometrial polyps are common in subfertile women and are associated with impaired fertility through multiple mechanisms, including mechanical interference, intrinsic molecular abnormalities, chronic inflammation, altered endometrial receptivity, and disruption of the immune microenvironment. Recent transcriptomic studies have revealed fundamental changes in gene expression in polyp tissue, including dysregulation of Wnt signaling and vascular smooth muscle genes, that may contribute to subfertility even in the absence of significant anatomical distortion.

Current evidence supports hysteroscopic polypectomy as an effective intervention to improve fertility outcomes, particularly in women undergoing intrauterine insemination. Level I evidence from randomized controlled trials demonstrates that polypectomy before IUI more than doubles pregnancy rates. For women undergoing IVF/ICSI, moderate-quality evidence suggests that polypectomy of documented polyps improves clinical pregnancy and live birth rates, though the magnitude of benefit is more variable.

Chronic endometritis frequently coexists with endometrial polyps and independently impairs fertility. Standardized diagnosis using CD138 immunohistochemistry with a threshold of ≥5 plasma cells per high-power field identifies women who benefit from antibiotic treatment. Doxycycline 100 mg orally twice daily for 14 days is the recommended first-line regimen, with test-of-cure biopsy to confirm resolution. Successful treatment of chronic endometritis substantially improves reproductive outcomes.

Clinical decision-making should be individualized based on polyp characteristics, patient age, fertility treatment plan, and patient preferences. Strong evidence supports polypectomy for symptomatic polyps, polyps ≥ 10 mm, and polyps in women undergoing IUI or IVF/ICSI. For small asymptomatic polyps (<10 mm), individualized decision-making is appropriate, considering the potential for spontaneous regression and the limited direct evidence for benefit of removal.

Future research should focus on identifying molecular predictors of polyp-associated infertility, optimizing the timing of polypectomy relative to fertility treatment, clarifying the role of polypectomy in specific subpopulations, and standardizing the diagnosis and treatment of chronic endometritis. These advances will enable more personalized and effective management of endometrial polyps in subfertile women.

## Figures and Tables

**Table 1:** Summary of Key Studies on Endometrial Polyps and Fertility Outcomes.

Study	Design	N	Intervention	Key Finding
Pérez-Medina 2005 [7]	RCT	215	Polypectomy vs. observation before IUI	PR 63.4% vs. 28.2% (RR 2.1, *p* < 0.001)
Triantafyllidou 2024 [15]	Retrospective cohort	40	Polypectomy in unexplained infertilitypatients	CPR 65% (26/40); total positive 72.5% (29/40)
Wang 2024 [16]	Meta-analysis	11,887	Hysteroscopy before IVF	CPR RR 1.34 (1.19–1.51); LBR RR 1.43 (1.21–1.69)
Nishioka 2023 [17]	Retrospective	156	Hysteroscopic vs. curettage polypectomy	PR 68.2% vs. 51.4% (OR 2.03)
Bougie 2024 [13]	Guideline (SOGC)	N/A	Polypectomy recommended for infertile women with polyps	Strong recommendation
Dunn 2018 [18]	Retrospective	389	Early vs. delayed ET after polypectomy	Early ET: lower LBR (38% vs. 51%, *p* = 0.02); delayed ET favored
Tuncer 2025 [14]	Retrospective	428	Risk factors for recurrence	Recurrence rate 7.78%
Wang 2024 [19]	Retrospective	186	GnRH-a pretreatment	LBR 53.3% vs. 43.3% (OR 1.47)

Abbreviations: RCT, randomized controlled trial; IUI, intrauterine insemination; PR, pregnancy rate; RR, relative risk; RIF, recurrent implantation failure; CPR, clinical pregnancy rate; IVF, in vitro fertilization; LBR, live birth rate; OR, odds ratio; CI, confidence interval; ET, embryo transfer; GnRH-a, gonadotropin-releasing hormone agonist; SOGC, Society of Obstetricians and Gynaecologists of Canada.

**Table 2:** Diagnostic Test Characteristics for Endometrial Polyp Detection.

Modality	Sensitivity (%)	Specificity (%)	Advantages	Limitations
2D-TVS	55–88	65–95	Non-invasive, widely available, low cost	Operator-dependent, limited in thick endometrium
3D-TVS	85–95	90–98	Better cavity visualization, multiplanar imaging	Requires specialized equipment, higher cost
SIS	87–94	81–94	Improved accuracy over TVS, office-based	Requires fluid instillation, mild discomfort
Hysteroscopy	92–100	75–94	Gold standard, allows simultaneous treatment	Invasive, requires instrumentation
MRI	80–90	85–95	No radiation, excellent soft tissue contrast	High cost, limited availability, not first-line

Abbreviations: 2D-TVS, two-dimensional transvaginal sonography; 3D-TVS, three-dimensional transvaginal sonography; SIS, saline infusion sonohysterography; MRI, magnetic resonance imaging.

**Table 3:** Pregnancy Outcomes After Polypectomy by Treatment Modality.

Treatment Modality	Clinical Pregnancy Rate (%)	Live Birth Rate (%)	Key Evidence	Level of Evidence †
Natural conception	28–65	22–55	Observational studies	III
IUI	51–63	43–55	Pérez-Medina 2005 RCT [7]	I
IVF/ICSI	53–72	43–66	Meta-analyses, cohort studies	I–II
RIF patients	45–73	38–62	Retospective cohorts	II–III

Abbreviations: IUI, intrauterine insemination; IVF, in vitro fertilization; ICSI, intracytoplasmic sperm injection; RIF, recurrent implantation failure. † Level of evidence: I = randomized controlled trial or meta-analysis of RCTs; II = prospective cohort study or meta-analysis of cohort studies; III = retrospective cohort or case–control study.

## Data Availability

No new data were created or analyzed in this study.

## Supplementary Materials

The following supporting information can be downloaded at: https://www.mdpi.com/article/10.3390/medicina62040692/s1, Table S1: Complete Included-Study List with Summary Data for All 83 Study Contributions; Supplementary Material S2: Database Search Strategies for PubMed/MEDLINE, Embase, Scopus, Web of Science, and Cochrane Library; Supplementary Material S3: PRISMA Flow Diagram of the Study Selection Process; Supplementary Material S4: SANRA (Scale for the Assessment of Narrative Review Articles) Quality Assessment Checklist.
