# Supplementary material for: Endometrial Polyps and Subfertility in Women Under 40: Pathophysiology, Fertility Outcomes, and Clinical Management
Source: Medicina (Kaunas). 2026 Apr 3;62(4):692. doi: 10.3390/medicina62040692 (PMC13117723; doi:10.3390/medicina62040692)
Supplement: Supplementary file 1 [file medicina-62-00692-s001.zip › medicina-4188208-supplementary/Clean_Supplementary_Materials.pdf]

Supplementary Materials

Endometrial Polyps and Subfertility in Women Under 40: Pathophysiology, Fertility Outcomes, and Clinical Management

Goksu Goc and Ozer Birge

Supplementary Material S2: Database Search Strategies

Search period: January 1, 2005 – December 31, 2024

A limited targeted update in January–March 2025 identified two additional studies (Pîrlog et al. 2025 and Wang et al. 2025); these are not included in the record totals below.

PubMed/MEDLINE (n = 612)

(("endometrial polyp"[MeSH Terms] OR "endometrial polyps"[Title/Abstract] OR "uterine polyp"[Title/Abstract] OR "intrauterine polyp"[Title/Abstract]) AND ("infertility"[MeSH Terms] OR "subfertility"[Title/Abstract] OR "infertility"[Title/Abstract] OR "fertility"[Title/Abstract] OR "pregnancy rate"[Title/Abstract] OR "conception"[Title/Abstract] OR "implantation"[Title/Abstract] OR "assisted reproductive technology"[Title/Abstract] OR "IVF"[Title/Abstract] OR "ICSI"[Title/Abstract] OR "IUI"[Title/Abstract] OR "in vitro fertilization"[Title/Abstract]))

Filters: Publication date 2005/01/01–2024/12/31; Species: Humans; Language: English

Embase via Ovid (n = 534)

1. exp endometrium polyp/ 2. (endometrial polyp* or uterine polyp* or intrauterine polyp*).ti,ab. 3. 1 or 2 4. exp infertility/ 5. exp subfertility/ 6. (infertil* or subfertil* or fertility or pregnancy rate* or conception or implantation).ti,ab. 7. exp assisted reproduction/ 8. exp in vitro fertilization/ 9. (IVF or ICSI or IUI or "assisted reproduct*" or "in vitro fertili*").ti,ab. 10. 4 or 5 or 6 or 7 or 8 or 9 11. 3 and 10 12. limit 11 to (human and english language and yr="2005-2024")

Scopus (n = 489)

TITLE-ABS-KEY(("endometrial polyp*" OR "uterine polyp*" OR "intrauterine polyp*") AND ("infertility" OR "subfertility" OR "fertility" OR "pregnancy rate" OR "implantation" OR "IVF" OR "ICSI" OR "IUI" OR "assisted reproductive technology" OR "in vitro fertilization")) AND PUBYEAR > 2004 AND PUBYEAR < 2025 AND LANGUAGE(english) AND DOCTYPE(ar OR re)

Web of Science Core Collection (n = 427)

TS=("endometrial polyp*" OR "uterine polyp*" OR "intrauterine polyp*") AND TS=(infertil* OR subfertil* OR "pregnancy rate*" OR "live birth" OR IVF OR ICSI OR IUI OR "assisted reproduct*") Refined by: Language: English; Document Types: Article OR Review; Timespan: 2005–2024

Cochrane Library / CENTRAL (n = 212)

#1 MeSH descriptor: [Polyps] explode all trees #2 MeSH descriptor: [Endometrium] explode all trees #3 #1 AND #2 #4 (endometrial polyp* OR uterine polyp* OR intrauterine polyp*):ti,ab,kw #5 #3 OR #4 #6 MeSH descriptor: [Infertility] explode all trees #7 MeSH descriptor: [Fertilization in Vitro] explode all trees #8 (infertil* OR subfertil* OR fertility OR "pregnancy rate*" OR IVF OR ICSI OR IUI):ti,ab,kw #9 #6 OR #7 OR #8 #10 #5 AND #9 #11 #10 with Cochrane Library publication date Between Jan 2005 and Dec 2024

Additional Sources (n = 78)

Reference list screening of included studies: 52 records. Citation searching of key articles (Pérez-Medina 2005, Wang 2024, SOGC 2024 guideline): 26 records.

Search Summary

Source	Records
PubMed/MEDLINE	612
Embase	534
Scopus	489
Web of Science	427
Cochrane Library	212
Reference screening + citation searching	78
**Total records identified**	**2,352**

Last search date: December 2024. Targeted update: January–March 2025 (2 additional studies).

Supplementary Material S3: PRISMA 2020 Study Selection Flow

Study selection following PRISMA 2020 guidelines.

Identification

Records identified through database searching: **2,274** (PubMed 612 + Embase 534 + Scopus 489 + Web of Science 427 + Cochrane 212)

Additional records from other sources: **78** (reference screening 52, citation searching 26)

Total records identified: **2,352**

Screening

Duplicates removed: **835**

Records screened (title and abstract): **1,517**

Records excluded at screening: **1,250**

Full-text articles assessed for eligibility: **267**

Full-Text Exclusion Reasons (n = 186)

Exclusion Reason	n
Not addressing fertility outcomes	67
Postmenopausal populations exclusively	34
Case reports with fewer than 10 patients	28
Non-English language	23
Conference abstracts without full text	18
Other reasons (duplicate cohorts, insufficient data)	16
**Total excluded at full text**	**186**

Verification: 67 + 34 + 28 + 23 + 18 + 16 = 186

Included Studies (n = 81 from database search + 2 targeted additions = 83 total)

Study Design	n
Randomized controlled trials	7
Systematic reviews and meta-analyses	12
Prospective cohort studies	14
Retrospective cohort studies	31

Case–control and other study designs	5
Narrative reviews and supporting evidence	11
Clinical guidelines and targeted 2025 additions	3
**Total included**	**81 from search + 2 targeted = 83**

Note: Two additional studies identified in the January–March 2025 targeted update (Pîrlog et al. 2025 and Wang et al. 2025) are included in the 83-study total but were identified outside the formal database search and are therefore not reflected in the 2,352-record count, the 267 full-text assessments, or the 186 exclusions above. The database search yielded 81 included studies; together with these 2 targeted additions, the final total is 83.

Supplementary Material S4: SANRA Quality Assessment

Scale for the Assessment of Narrative Review Articles (SANRA). Each item scored 0–2 (0 = low, 1 = moderate, 2 = high). Maximum: 12.

Reference: Baethge C, Goldbeck-Wood S, Mertens S. Res Integr Peer Rev. 2019;4:5.

SANRA Item	Score	Justification
1. Justification of importance	**2**	Introduction establishes clinical significance with prevalence data, economic implications, and unresolved controversies
2. Statement of aims	**2**	Six specific aims listed in Introduction covering epidemiology, pathophysiology, diagnosis, outcomes, CE, and clinical guidance
3. Literature search description	**2**	Section 2.1–2.3: 5 databases, date range, search terms, inclusion/exclusion criteria. Full strategies in S2
4. Referencing	**2**	110 references cited. Recent high-quality studies (2020–2025) prioritized. Key claims supported by specific citations
5. Scientific reasoning	**2**	Evidence quality discussed per section. RCT vs observational distinguished. Subgroup analysis limitations noted
6. Appropriate presentation	**2**	3 tables, 2 figures, 5 supplementary materials. Data presented with effect sizes and confidence intervals
**TOTAL**	**12/12**	**Excellent quality**

Supplementary Material S1 and Supplementary Table S1

The complete included-study list with summary data for all 83 study contributions is provided in the accompanying file: ***Supplementary_Table_S1.docx***

This file contains:

• Complete bibliographic details for all 83 study contributions, organized by study design

• Included-study summary columns: author(s), year, journal, study design, sample size, main finding/focus, and reference number

• Reference numbers corresponding to the main manuscript reference list
