# Supplementary material for: Endometrial Polyps and Subfertility in Women Under 40: Pathophysiology, Fertility Outcomes, and Clinical Management
Source: Medicina (Kaunas). 2026 Apr 3;62(4):692. doi: 10.3390/medicina62040692 (PMC13117723; doi:10.3390/medicina62040692)
Supplement: Supplementary file 1 [file medicina-62-00692-s001.zip › medicina-4188208-supplementary/Clean_Supplementary_Table_S1.pdf]

Supplementary Table S1: Complete Included-Study List with Summary Data

This supplementary table provides a complete included-study list with summary data for all 83 study contributions, organized by study design. Reference numbers in brackets [#] correspond to the main manuscript reference list. Studies not directly cited are marked with an asterisk (*). Studies published before the search window (2005–2024) identified through reference/citation screening are marked with a dagger (†).

‡ = Published online in late 2024/early 2025; captured in the main database search (within the 2,352-record total).

‡‡ = Identified in the January–March 2025 targeted update; NOT included in the 2,352-record total.

§ = Same publication as entry #3 (Shokeir 2004); listed separately because it contributes both RCT-level polypectomy outcome data and case-control prevalence data. This dual listing accounts for 83 study contributions from 82 unique publications.

1. RANDOMIZED CONTROLLED TRIALS (n = 7)

No.	First Author, Year	Journal	Design	N	Main Finding / Focus	Ref
1	Pérez-Medina T, 2005	Hum. Reprod.	RCT	215	Polypectomy before IUI: PR 63% vs 28% (RR 2.1, 95% CI 1.5–2.9, p<0.001). LANDMARK STUDY	[7]
2	Campo R, 2005	Hum. Reprod.	RCT	1,046	Office hysteroscopy success factors: 97% completion rate	*
3	Shokeir TA, 2004 †	J. Obstet. Gynaecol. Res.	RCT	204	Polypectomy improved pregnancy: 63% vs 28% (p<0.001). Also case-control prevalence analysis (see #66)	[17]
4	Varasteh NN, 1999 †	Obstet. Gynecol.	RCT	215	Pregnancy after polypectomy: 78.3% vs controls	[5]
5	Amer MI, 2010	J. Minim. Invasive Gynecol.	RCT	101	Office polypectomy vs expectant: 51.4% vs 25.0% pregnancy	*
6	Yanaihara A, 2008	Fertil. Steril.	RCT	89	Polypectomy before ART: improved implantation (19.6% vs 12.3%). Also case-control analysis (see #67)	[28]
7	Elsedeek MS, 2008	Arch. Gynecol. Obstet.	RCT	156	Polypectomy in infertility: 48.7% vs 16.7% pregnancy (p<0.001)	*

2. SYSTEMATIC REVIEWS AND META-ANALYSES (n = 12)

No.	First Author, Year	Journal	Design	N	Main Finding / Focus	Ref
8	Bosteels J, 2018	Cochrane Database Syst. Rev.	SR	3,246	Cochrane: hysteroscopy for subfertility, moderate quality evidence	[8]
9	Lieng M, 2010	Acta Obstet. Gynecol. Scand.	SR	1,892	Treatment of endometrial polyps: comprehensive review	[3]
10	Salim S, 2011	J. Minim. Invasive Gynecol.	SR	2,104	Diagnosis and management: critical review	[1]
11	Pereira N, 2015	Surg. Res. Pract.	SR	1,456	Surgical management in infertile women	*
12	Ludwin A, 2020	Ultrasound Obstet. Gynecol.	MA	25 studies	SIS vs 2D-TVS: SIS superior (sensitivity 95%, specificity 94%)	[62]
13	Kaveh M, 2020	Videosurgery Miniinvasive Tech.	MA	18 studies	SIS, TVS, hysteroscopy comparison	*
14	Zhang Y, 2019	J. Minim. Invasive Gynecol.	SR	5 studies	Polypectomy vs no treatment in ART: improved outcomes (OR 2.9)	[74]
15	Modesto W, 2020	Int. J. Reprod. Contracept. Obstet. Gynecol.	MA	6 studies	Polypectomy vs expectant in asymptomatic: benefit shown	*
16	Afifi K, 2010	Eur. J. Obstet. Gynecol. Reprod. Biol.	SR	12 studies	Management in subfertile women	*
17	Mouhayar Y, 2017	Eur. J. Obstet. Gynecol. Reprod. Biol.	Cost + SR	—	Cost-effective: polypectomy saves costs per pregnancy in IUI/IVF	[86]
18	Craciunas L, 2019	Hum. Reprod. Update	MA	—	Endometrial receptivity markers: systematic review	*
19	Fiore A, 2025 ‡	Reprod. Biomed. Online	MA	—	Association of endometriosis and endometrial polyps	*

3. PROSPECTIVE COHORT STUDIES (n = 14)

No.	First Author, Year	Journal	Design	N	Main Finding / Focus	Ref
20	Wen W, 2024	J. Army Med. Univ.	Prospective	388	Polyps with ART: comparable outcomes after polypectomy	[79]
21	Al-Quran FA, 2018	J. R. Med. Serv.	Prospective	150	Spontaneous pregnancy after polypectomy: 28%	*
22	Yang JH, 2017	Reprod. Biomed. Online	Prospective	168	Incidental polyps during IVF: 63% vs 41% pregnancy	*
23	Nomiyama M, 2021	Reprod. Med. Biol.	Prospective	118	Polyps with plasma cells: CE association, 42.4% pregnancy	*
24	Kahraman K, 2016	Clin. Exp. Obstet. Gynecol.	Prospective	83	Polyp size effect: ≤1cm 44.4%, >1cm 48.9% (NS)	*
25	Oral B, 2022	J. Turk. Soc. Obstet. Gynecol.	Prospective	156	Polypectomy during COH: no cycle cancellation needed <1.5cm	*
26	Cicinelli E, 2005	Hum. Reprod.	Prospective	438	Micropolyps suggest chronic endometritis	[12]
27	Johnston-MacAnanny EB, 2010	Fertil. Steril.	Prospective	95	CE in RIF: 30% prevalence	[43]
28	Kasius JC, 2011	Fertil. Steril.	Prospective	385	CE impact: OR 0.44 for ongoing pregnancy	[90]
29	Kitaya K, 2017	Am. J. Reprod. Immunol.	Prospective	85	Antibiotic for CE: improved live birth rate	[87]
30	McQueen DB, 2014	Fertil. Steril.	Prospective	127	CE in RPL: 27% prevalence	[88]
31	Bettocchi S, 2004 †	Obstet. Gynecol. Clin. N. Am.	Prospective	1,000+	Office hysteroscopy: feasibility and safety	[65]
32	Güven MA, 2004 †	Int. J. Gynaecol. Obstet.	Prospective	230	Hydrosonography screening: 87% sensitivity for polyps	[72]
33	Ayida G, 1997 †	Ultrasound Obstet. Gynecol.	Prospective	102	Cavity assessment before IVF: SIS vs hysteroscopy	[73]

4. RETROSPECTIVE COHORT STUDIES (n = 31)

No.	First Author, Year	Journal	Design	N	Main Finding / Focus	Ref
34	Triantafyllidou O, 2024	J. Clin. Med.	Retrospective	40	Polypectomy in unexplained infertility + IVF: CPR 65% (26/40)	[78]
35	Wang CW, 2024	Taiwan. J. Obstet. Gynecol.	Retrospective	660	GnRH-a after multiple polypectomies: improved outcomes	[85]
36	Nishioka Y, 2023	Medicina	Retrospective	434	Hysteroscopy vs curettage: 68.2% vs 51.4% pregnancy (OR 2.03)	[77]
37	Dunn RC, 2018	Fertil. Steril.	Retrospective	389	Early (<30d) vs delayed ET after polypectomy: early lower LBR	[81]
38	Tiras B, 2012	Reprod. Biomed. Online	Retrospective	94	Polyps before/during ICSI: 29.8% vs 38.3% pregnancy (NS)	*
39	Karasmanoglu AD, 2016	Med. Sci. Monit.	Retrospective	256	Hysteroscopic findings in infertile women: polyps 18.4%	*
40	Niknejadi M, 2012	Iran. J. Radiol.	Retrospective	178	TVS diagnostic accuracy: 88.3% sensitivity	*
41	Borges R, 2015	Womens Health	Retrospective	156	TVS vs hysteroscopy: hysteroscopy superior	*
42	Chami R, 2021	J. Gynecol. Obstet. Hum. Reprod.	Retrospective	—	Endometrial polyps and subfertility: comprehensive review	*
43	Vaduva CC, 2022	Rom. J. Morphol. Embryol.	Retrospective	234	Polyps, CE, endometriosis association	*
44	Unlu C, 2016	Reprod. Sci.	Retrospective	41	HOXA10/11 increase after myomectomy: 12.8-fold, 9.0-fold	*
45	Jiang NX, 2017	Cell Death Discov.	Retrospective	89	HOXA10 sumoylation in RIF: novel mechanism	*
46	Adamczyk M, 2022	Int. J. Mol. Sci.	Retrospective	67	Epigenetic factors in endometrium with endometriosis	*
47	Ben-Nagi J, 2009	Reprod. Biomed. Online	Retrospective	619	Endometrial implantation factors with submucous fibroids	*
48	Dreisler E, 2009	Ultrasound Obstet. Gynecol.	Retrospective	250	Polyp prevalence increases with age: 8% by age 40	[2]
49	Svirsky R, 2008	Eur. J. Obstet. Gynecol. Reprod. Biol.	Retrospective	678	Polyp location and fertility: fundal polyps worse outcomes	*
50	Hinckley MD, 2004 †	JSLS	Retrospective	1,000	1000 office hysteroscopies prior to IVF	[33]
51	Spiewankiewicz B, 2003 †	Clin. Exp. Obstet. Gynecol.	Retrospective	89	Effectiveness of hysteroscopic polypectomy in infertility	[32]
52	Lass A, 1999 †	J. Assist. Reprod. Genet.	Retrospective	452	Effect of polyps on IVF outcomes	[29]
53	Rackow BW, 2011	Fertil. Steril.	Retrospective	562	Endometrial polyps affect uterine receptivity	[7]
54	Silberstein T, 2006	Isr. Med. Assoc. J.	Retrospective	234	Endometrial polyps in fertile and infertile women	[31]
55	Di Spiezio Sardo A, 2016	Eur. J. Obstet. Gynecol. Reprod. Biol.	Retrospective	398	Prevention of intrauterine adhesions: systematic review	[9]
56	Eldar-Geva T, 1998 †	Fertil. Steril.	Retrospective	687	Effect of fibroids on ART outcomes	*
57	Shohayeb A, 2011	Fertil. Steril.	Retrospective	178	Submucous myomas and pregnancy rates	*
58	Richlin SS, 2002 †	Hum. Reprod.	Retrospective	64	Glycodelin levels in polyps: implications for implantation	[6]
59	Marwood M, 2009	Endocrinology	Retrospective	—	IL-11 and LIF regulate endometrial epithelial adhesion	*

60	Rshoud H, 2019	Middle East Fertil. Soc. J.	Retrospectiv e	78	Polyp removal: natural conception and IUI improved	*
61	Tuncer SF, 2025 ‡	Med. Sci. Monit.	Retrospectiv e	—	Risk factors for recurrence after polypectomy	*
62	Savelli L, 2009	Ultrasound Obstet. Gynecol.	Retrospectiv e	—	TVS for assessment of pelvic endometriosis	*
63	Kara M, 2019	Gynecol. Minim. Invasive Ther.	Retrospectiv e	—	HOXA-10, HOXA-11, LIF in PCOS patients	*
64	Stamatellos I, 2008	Arch. Gynecol. Obstet.	Retrospectiv e	83	Polyp size/number effect on pregnancy rates	[35]

5. CASE-CONTROL AND OTHER STUDY DESIGNS (n = 5)

No.	First Author, Year	Journal	Design	N	Main Finding / Focus	Ref
65	Shokeir TA, 2004 †	J. Obstet. Gynaecol. Res.	Case-Control	204	Case-control component: polyps in infertile vs fertile 26% vs 3%	[17]§
66	Yanaihara A, 2008	Fertil. Steril.	Case-Control	178	Case-control component: polyps and implantation failure OR 2.8 for RIF	[30]
67	Shushan A, 2004 †	Gynecol. Obstet. Invest.	Case-Control	198	Tamoxifen and polyps: increased risk in breast cancer patients	[26]
68	Taylor HS, 1998 †	J. Clin. Invest.	Lab/Mechanistic	—	HOXA10 expression at implantation: sex steroid response	[46]
69	Ashary N, 2020	Int. J. Dev. Biol.	Narrative Review	—	Homeobox genes: development to decidualization	*

6. NARRATIVE REVIEWS AND SUPPORTING EVIDENCE (n = 11)

No.	First Author, Year	Journal	Design	N	Main Finding / Focus	Ref
70	Vitale SG, 2021	Eur. J. Obstet. Gynecol. Reprod. Biol.	Evidence Guide	—	Evidence-based diagnosis and management guide	*
71	Jee BC, 2021	Clin. Exp. Reprod. Med.	Mini-review	—	Management of endometrial polyps in infertile women	*
72	Lessey BA, 2017	Fertil. Steril.	Review	—	Endometrial receptivity in endometriosis	*
73	Orazov MR, 2023	Medical Council	Review	—	Endometrial receptivity in adenomyosis	*
74	Bai F, 2021	Reprod. Biol. Endocrinol.	SR	—	Endometrial receptivity in PCOS: systematic review	[24]
75	Guo SW, 2018	Hum. Reprod. Update	Review	—	Paradigm shift in endometriosis/adenomyosis research	*
76	Guo SW, 2018	Reprod. Biomed. Online	Review	—	Dating endometrial biopsies: methodological critique	*
77	Navarro A, 2021	Front. Cell Dev. Biol.	Review	—	Uterine fibroids: impact on endometrium function	*
78	Celik O, 2015	Fertil. Steril.	Lab study	—	Endometrioma resection increases HOXA-10/11 expression	*
79	Du H, 2016	Cold Spring Harb. Perspect. Med.	Review	—	Role of Hox genes in female reproductive tract	*
80	Friedler S, 1993 †	Hum. Reprod.	Review	—	Incidence of post-abortion intrauterine adhesions	*

7. CLINICAL GUIDELINES AND TARGETED 2025 ADDITIONS (n = 3)

No.	First Author, Year	Journal	Design	N	Main Finding / Focus	Ref
81	Bougie O (SOGC), 2024	J. Obstet. Gynaecol. Can.	Clinical Guideline	N/A	SOGC guideline on diagnosis and management of polyps	[14]
82	Pîrlog LM, 2025 ‡‡	Biomolecules	Review (Targeted)	—	HOXA10/HOXA11 in endometrial benign disorders	[47]
83	Wang C, 2025 ‡‡	BMC Surg.	Retrospective (Targeted)	240	Manual tissue removal vs conventional resection in IVF	[84]

Verification

Category totals: 7 + 12 + 14 + 31 + 5 + 11 + 3 = 83 study contributions (82 unique publications).

Unique publications: 82 (Shokeir 2004 appears as entries #3 and #65).

2025 studies: Fiore 2025 (#19, ‡) and Tuncer 2025 (#61, ‡) were captured in the main database search. Pîrlog 2025 (#82, ‡‡) and Wang C 2025 (#83, ‡‡) were identified in the targeted January–March 2025 update and are not included in the 2,352-record search total.
